# Specific quantitative detection of *Streptococcus suis* and *Actinobacillus pleuropneumoniae* in co-infection and mixed biofilms

**DOI:** 10.3389/fcimb.2022.898412

**Published:** 2022-08-03

**Authors:** Li Yi, Manyu Jin, Mengxia Gao, Haikun Wang, Qingying Fan, Daniel Grenier, Liyun Sun, Shaohui Wang, Yang Wang

**Affiliations:** ^1^ College of Life Science, Luoyang Normal University, Luoyang, China; ^2^ Shanghai Veterinary Research Institute, Chinese Academy of Agricultural Sciences, Shanghai, China; ^3^ College of Animal Science and Technology, Henan University of Science and Technology, Luoyang, China; ^4^ Groupe de Recherche en Écologie Buccale (GREB), Faculté de Médecine Dentaire, Université Laval, Quebec City, QC, Canada

**Keywords:** *Streptococcus suis*, *Actinobacillus pleuropneumoniae*, TaqMan real-time PCR, co-infection, biofilm

## Abstract

Respiratory infections seriously affect the swine industry worldwide. Co-infections of two vital pathogenic bacteria *Streptococcus suis* (*S. suis*) and *Actinobacillus pleuropneumoniae* (*A. pleuropneumoniae*), colonizing the respiratory tract often occurs in veterinary clinical practice. Moreover, our previous research found that *S. suis* and *A. pleuropneumoniae* can form biofilm *in vitro*. The formation of a mixed biofilm not only causes persistent infections, but also increases the multiple drug resistance of bacteria, which brings difficulties to disease prevention and control. However, the methods for detecting *S. suis* and *A. pleuropneumoniae* in co-infection and biofilm are immature. Therefore, in this study, primers and probes were designed based on the conservative sequence of *S. suis gdh* gene and *A. pleuropneumoniae apx*IVA gene. Then, a TaqMan duplex real-time PCR method for simultaneous detection of *S. suis* and *A. pleuropneumoniae* was successfully established *via* optimizing the reaction system and conditions. The specificity analysis results showed that this TaqMan real-time PCR method had strong specificity and high reliability. The sensitivity test results showed that the minimum detection concentration of *S. suis* and *A. pleuropneumoniae* recombinant plasmid was 10 copies/μL, which is 100 times more sensitive than conventional PCR methods. The amplification efficiencies of *S. suis* and *A. pleuropneumoniae* were 95.9% and 104.4% with R^2^ value greater than 0.995, respectively. The slopes of the calibration curves of absolute cell abundance of *S. suis* and *A. pleuropneumoniae* were 1.02 and 1.09, respectively. The assays were applied to cultivated mixed biofilms and approximately 10^8^ CFUs per biofilm were quantified when 10^8^ CFUs planktonic bacteria of either *S. suis* or *A. pleuropneumoniae* were added to biofilms. In summary, this study developed a TaqMan real-time PCR assay for specific, accurate quantification of *S. suis* or *A. pleuropneumoniae* in mixed biofilms, which may help for the detection, prevention and control of diseases caused by a bacterial mixed infection involving *S. suis* and *A. pleuropneumoniae*.

## Introduction

Respiratory system diseases seriously affect the development of the swine industry worldwide. *Streptococcus suis* (*S. suis*) and *Actinobacillus pleuropneumoniae* (*A. pleuropneumoniae*) are two important respiratory pathogens in the swine industry (Opriessnig et al., 2011). *S. suis* is a zoonotic pathogen that can cause serotype-dependent diseases in swine and humans, mainly as meningitis (Weinert et al., 2015; Segura et al., 2017). *A. pleuropneumoniae* is the etiologic agent of porcine contagious pleuropneumonia (PCP) disease in swine, which is considered to be one of the leading causes of economic losses in the swine industry (Mullebner et al., 2018). Meanwhile, *S. suis* and *A. pleuropneumoniae* are major pathogens of the Porcine Respiratory Disease Complex (PRDC) (Opriessnig et al., 2011). Clinically, *S. suis* and *A. pleuropneumoniae* infections are often detected simultaneously. *in vitro*Biofilm is a structured surface-bound community formed by microorganisms (Kolter and Greenberg, 2006). Most of the biofilms exist in the form of mixed-species communities (Costerton et al., 1999; Elias and Banin, 2012). The complex interspecies dynamics modulate the development, nature, and survival of biofilms and will affect the pathological process and treatment outcome of biofilm-associated infections, especially by enhancing resistance to host immune defense (Rendueles and Ghigo, 2012; Burmolle et al., 2014). Once the pathogens form a biofilm in the host, they often cause persistent infections and are rather difficult to eliminate (Figueiredo et al., 2017; Wang et al., 2018). In some cases, microbial interactions in mixed biofilms can provide conditions for the exchange of resistance genes or increased survival (Horter et al., 2001; Harriott and Noverr, 2009). In addition, our studies found that *S. suis* and *A. pleuropneumoniae* can form two-species biofilms when co-cultured *in vitro*. When grown in dual-species biofilms *in vitro*, the genes related to virulence factors in *S. suis* and *A. pleuropneumoniae* are significantly up-regulated. Compared with monocultures, the antibiotic resistance of *S. suis* and *A. pleuropneumoniae* is both enhanced in the co-culture model (Wang et al., 2020). The mixed biofilm formed by *S. suis* and *A. pleuropneumoniae* leads to the increase of drug resistance, which increases the difficulties of disease prevention and control. Furthermore, the detection methods of the mixed biofilm formed by the two bacteria are seldom reported. Therefore, it is urgent to invest efforts and time on investigating mixed biofilms and developing tools for such studies.

Efficient and accurate detection methods play a vital role in the effective prevention and control of the disease. However, traditional diagnosis has some disadvantages in practical application, such as complex cultivation methods, time-consuming and low sensitivity, which cannot quantitatively analyze the pathogenic bacteria in the biofilm. Real-time polymerase chain reaction (PCR) is a powerful and easy to use technology that represents an ideal method for quantifying species in biofilms. Real-time PCR provides high levels of specificity and overcomes some limitations of conventional bacteria quantification methods, because it distinguishes morphologically-similar species by specifically targeting genes of a single species. The real-time PCR assays mainly include the dye method represented by SYBR Green real-time PCR and the probe method represented by TaqMan real-time PCR. SYBR Green real-time PCR uses a fluorescent dye that can bind to any DNA. Whereas, TaqMan real-time PCR uses probe dyes that can specifically bind to target DNA. The specificity of TaqMan real-time PCR is crucial for quantifying the number of individual species in a mixed biofilm (Kirakodu et al., 2008; Cao and Shockey, 2012). Thus, TaqMan real-time PCR should be considered as an ideal method for quantifying bacterial pathogens embedded in a mixed biofilm (Suzuki et al., 2005).

In order to accurately and specifically quantify the clinical co-infection and mixed biofilm formed by *S. suis* and *A. pleuropneumoniae*, we developed a specific TaqMan real-time PCR assay that can simultaneously quantify *S. suis* and *A. pleuropneumoniae*. The real-time PCR method was evaluated for its specificity, sensitivity, application for detection and diagnosis. This study provides an efficient real-time PCR tool available for the quantify detection for *S. suis* and *A. pleuropneumoniae* in co-infection and biofilms, helping for the prevention and control of diseases caused by these two important respiratory pathogens.

## Materials and methods

### Bacterial strains and growth conditions

*S. suis* serotype 2 strain HA9801 was isolated from an infected pig in Jiangsu Province and confirmed to be a pathogenic strain in pig model as previously description (Yao et al., 1999). *A. pleuropneumoniae* CVCC265 (APP) (serotype 1) was purchased from China Veterinary Culture Collection Center (CVCC). *S. suis* was grown in Tryptic Soy Broth (TSB) or Tryptic Soy Agar (TSA) medium for 12 hours, then diluted with 1:100 and placed in 37°C shaking table at 180 r/min for 8 hours. *A. pleuropneumoniae* was grown in TSB or TSA supplemented with 0.1 μg/mL of β-nicotinamide-adenine-dinucleotide (NAD) for 18 hours, then dilute it with 1:100 and place it in a 180 r/min 37°C shaking table for 10 hours.

### Primer and probe design

The sequences of the *S. suis gdh* gene and *A. pleuropneumoniae apx*IVA gene (GenBank ID: AY853916 and AF021919) were aligned and analyzed using DNASTAR 7.10. Primers and probes (**Table 1**) for conventional PCR and TaqMan real-time PCR were designed using prime premier 5.0 software. PCR amplification, electrophoresis and sequencing verified that the designed primers can amplify the target sequence.

**Table 1:** Primers and probes used in this study.

Primer/Probe	Sequences (5’ - 3’)^a^	Amplicon length (bp)
SS-gdh-QF	GTCATGGACTCGTGAAGAAGTAG	106
SS-gdh-QR	GTAGTCTGTACCAAGGTCGTATTT	
SS-gdh-QP	FAM-TTGGCTGTGTTGAAGATGTTGGCC-BHQ2	
APP-apxIVA-QF	GGTGGAACGGTAAACCTTAACT	118
APP-apxIVA-QR	CTTTCGCCGCATTCACTAAAC	
APP-apxIVA-QP	Texas Red-AGGTGGAAACCTACACGTTAGACGA-BHQ2	
SS-gdh-F	GTTGAGCCTGAGCGTATCATC	425
SS-gdh-R	CCAGTCAAGACACCTGCATC	
APP-16S rRNA -F	GGAGCTTGCTTTCTTTGCCGACG	826
APP-16S rRNA -R	TAACCTTGCGGCCGTACTCCC	

a BHQ, black hole quencher; FAM, 6-carboxyfluorescein; Texas Red, a derivative of Texas Red sulfonyl chloride.

### DNA extraction and construction of recombinant plasmid

DNA extraction from 3 mL bacterial culture solution was performed using the TIANamp Bacteria DNA Kit according to the manufacturer’s instructions with a final elution volume of 50 μL. The concentration and purity of the extracted DNA samples were determined by agarose gel electrophoresis and NanoDrop 2000C (Thermo Fisher Scientific Inc.) spectrophotometry. Genomic DNA of SS and APP strains were used as templates. Target genes of the two strains were amplified by PCR using corresponding primers (**Table 1**) and inserted into pMD19-T vector. The resultant recombinant plasmids (pMD-gdh and pMD-apxIVA) were transformed into *Escherichia coli* DH5α, respectively. The positive recombinant plasmid was sequenced by Sangong Bioengineering (Shanghai) Co., LTD., and used as the plasmid standard of the corresponding bacteria.

### TaqMan real-time PCR reactions

TaqMan real-time PCR amplification was performed using a Bio-Rad CFX96 Touch real-time PCR instrument. Using the recombinant plasmids pMD-gdh and pMD-apxIVA as templates, the real-time PCR amplifications were conducted using triplicate samples, three no-template controls, and 4 inter-plate calibrators in duplicate. Conventional PCR reaction system: total system 20 μL, 2 × PCR Master Mix 10 μL, 1 μL of upstream and downstream primers, 2 μL of template DNA, ddH_2_O to obtain a final volume of 20 μL; reaction conditions: pre-denaturation at 95 °C for 5 minutes, denaturation at 95 °C; for 30 s, annealing at 55 °C; for 30 s, extension at 72 °C; for 60 s, for 35 cycles, re-extension at 72 °C; for 10 minutes.

### Specificity and cross-reactivity

The specificity of the TaqMan real-time PCR assay was evaluated using bacterial swine pathogens causing respiratory diseases as well as bacteria prevalent in the animal environment. TaqMan real-time PCR was used to amplify the genomic DNA of *S. suis*, *A. pleuropneumoniae*, *Bordetella bronchiseptica*, *Staphylococcus aureus*, *Pseudomonas aeruginosa*, *Haemophilus parasuis*, *Pasteurella multocida* and *Streptococcus agalactiae.*

The real-time PCR primer/probe sets were tested for cross reactivity between *S. suis* and *A. pleuropneumoniae*. On the one hand, 10-10^7^ copies/μL of *A. pleuropneumoniae* recombinant plasmids were subjected to TaqMan real-time PCR amplification using SS-gdh-QF/R and SS-gdh-QP. On the other hand, 10-10^7^ copies of *S. suis* recombinant plasmids were subjected to TaqMan real-time PCR amplification using APP-apxIVA-Q/R and APP-apxIVA-QP.

### Standard curve determination of TaqMan real-time PCR

The TaqMan real-time PCR standard curve of *S. suis* and *A. pleuropneumoniae* can be plotted based on Cq value and Log_10_ copy number of 10-fold gradient dilution of recombinant plasmids. Plasmid abundance was calculated by equation (Whelan et al., 2003). 


$$C_{molecules}=\frac{\;C_{mass}\cdot N_{A}}{L_{plasmid\;}\cdot M_{basepair}\;}$$

C_mass_ is the concentration of the plasmid (ng/μL), L_plasmid_ is the length of the plasmid (bp), M_basepair_ is the mass of base pairs assumed to be 660 Da basepairs ^-1^, and N_A_ is the Avogadro’s Constant.

Recombinant plasmids were extracted and their concentration and purity were determined using NanoDrop 2000C ultra-micro spectrometer. The recombinant plasmid was converted into target gene copy number and 10-fold diluted in the range of 10^3^ to 10^7^ copies, which were used as template of TaqMan real-time PCR, respectively. Average Cq values were plotted against the Log_10_ copy number to calculate the efficiency, slope and y-intercept of each standard curve and obtain a linear equation. Standard curves with an efficiency of 90%-110% were deemed suitable for real-time PCR analysis.

### Assessment of sensitivity and repeatability

The recombinant plasmid of *S. suis* and *A. pleuropneumoniae* were ten-fold serially diluted to 1×10^4^ copies/μL ~1 copy/μL. The optimized reaction system and conditions were used for TaqMan real-time PCR amplification with serially dilutions of recombinant plasmid and ddH_2_O as templates to evaluate the sensitivity of the detection method established in this study.

The repeatability of TaqMan real-time PCR was evaluated using recombinant plasmid mixture of *S. suis* and *A. pleuropneumoniae*, which were ten-fold serially diluted to 1×10^6^ copies/μL~1×10^4^ copies/μL. Repeatability within the group: amplification of the dilutions was performed by TaqMan real-time PCR in triplicate. For assessing the repeatability between groups, amplification of dilutions was performed by TaqMan real-time PCR for three consecutive weeks at intervals of one week. The coefficient of variation was calculated using Excel 2016 software (Microsoft).

### Calibration curve validation

A bacterial calibration curve was built based on the copy number of the target gene quantified by TaqMan real-time PCR and the bacterial concentration of ten-fold serial dilutions. Bacteria cultured overnight were quantified to 10^8^ colony forming units (CFUs), and then ten-fold serially diluted to bring the number of bacteria to 10^6^-10^2^ CFUs. The number of bacterial CFUs was confirmed by plating on TSA plates. DNA was extracted from the samples according to the method described in section 2.2 and quantified using established TaqMan real-time PCR reactions.

### Quantification of *S. suis* and *A. pleuropneumoniae* in clinical samples

To evaluate the discrimination and applicability of established method for clinical samples, a total of 45 lung samples from porcine suspected to be infected with *S. suis* and *A. pleuropneumoniae* were collected from farms in Henan Province, China. Lung tissues were minced by mill, diluted 1:10 in phosphate-buffered saline (PBS, pH 7.4), and genomic DNA of the samples was extracted according to the procedure described above. The genomic DNA extracted from lung tissue samples, the mixed genomic DNA of *S. suis* and *A. pleuropneumoniae*, and ddH_2_O were used as templates. The TaqMan real-time PCR and conventional PCR were used for detection, and the experimental results were statistically analyzed and compared.

### Quantification of *S. suis* and *A. pleuropneumoniae* biofilm samples *in vitro*

First, the mixed biofilm samples of *S. suis* and *A. pleuropneumoniae* were cultrued. Pure cultures (bacterial culture medium) of *S. suis* and *A. pleuropneumoniae* were grown to the exponential phase (OD600, 0.6-0.7) and used to inoculate 24-well polystyrene, flat bottom, tissue culture-treated microplates in a ratio of 1:1 and a final volume added to each well of 2 mL. The microplates were then incubated at 37°C for 48-72 h.

In order to evaluate the effectiveness of the established TaqMan real-time PCR assays in a biofilm system, biofilm samples were spiked with *S. suis* and *A. pleuropneumoniae* cultures, and then analyzed by real-time PCR method. Four different samples were created in triplicate using a 24-well plate method: i) mixed biofilm, ii) mixed biofilm + 10^8^ CFUs pure culture of *S. suis*, iii) mixed biofilm + 10^8^ CFUs pure culture of *A. pleuropneumoniae*, and iv) mixed biofilm + 10^8^ CFUs pure culture of *S. suis* + 10^8^ CFUs pure culture of *A. pleuropneumoniae*. Culture biofilm according to the above method. The cultured biofilms were washed three times with PBS, and 200 μL of PBS was added to each well, followed by ultrasonic treatment with an ultrasonic cleaner for 5 min, and finally the amount of each group of samples was adjusted to 200 μL. DNA was extracted from the samples according to the procedure described above, and the DNA samples were amplified by TaqMan real-time PCR. In addition, the biofilm diluent of *S. suis* and *A. pleuropneumoniae* were diluted 10-fold with PBS to 10^2^-10^6^ CFUs/mL biofilm diluent, and the biofilm diluent was amplified by TaqMan real-time PCR to determine the minimum detection limit of biofilm state by this method. Cell abundance was calculated using the developed calibration curves.

### Statistical analysis

Quantitative variables were expressed as means ± standard error (SE). Calibration curves were calculated using linear regression with 95% confidence intervals. All calculations and plots were completed using GraphPad v7.

## Results

### Establishment and optimization of TaqMan real-time PCR assay

Based to the bioinformatics analysis, in order to improve the amplification efficiency, we worked on improving the specificity and stability of the primer as well as probes. We optimized the annealing temperature, primer and probe concentration. The final optimal conditions were as follows: 95°C for 2 minutes, followed by 40 cycles of 95°C for 15 s, 60°C for 30 s. Amplification reactions were performed in a 20 µL reaction volume involving of 10 µL Bestar^®^ 2 × qPCR Master Mix, 0.6 μL of each primers (0.3 μM), 0.3 μL of probes (0.15 μM), 1 μL of template DNA, and ddH_2_O to obtain a final volume of 20 μL. Under these optimization conditions, the *S. suis* and *A. pleuropneumoniae* were specifically detected by TaqMan duplex real-time PCR assay.

### Specificity analysis and cross-reactivity of TaqMan real-time PCR assay

**Figure 1:**
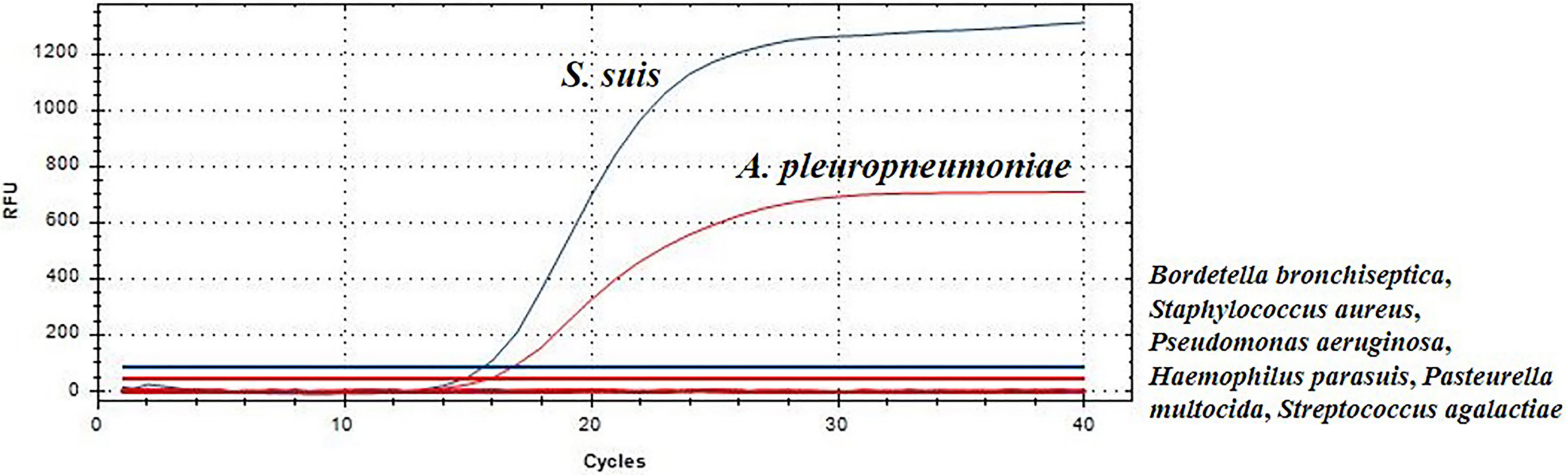
Evaluation of specificity of TaqMan real-time PCR.

To analyze the specificity of this TaqMan real-time PCR, *S. suis*, *A. pleuropneumoniae*, *Bordetella bronchiseptica*, *Staphylococcus aureus*, *Haemophilus parasuis*, *Pseudomonas aeruginosa*, *Pasteurella multocida* and *Streptococcus agalactiae*, were used as templates for TaqMan real-time PCR, respectively. *S. suis* strain HA9801 and *A. pleuropneumoniae* strain CVCC265 showed a fluorescent signal (**Figure 1**). Moreover, other serotypes of *S. suis* and *A. pleuropneumoniae* strains also yielded positive results in this TaqMan real-time PCR. Whereas, other bacteria yielded negative results in the duplex real-time PCR, indicating that the TaqMan real-time PCR method is specific. These experiments validate the real-time PCR assays abilities to specifically distinguish *S. suis* and *A. pleuropneumoniae*.

### Standard curves

**Figure 2:**
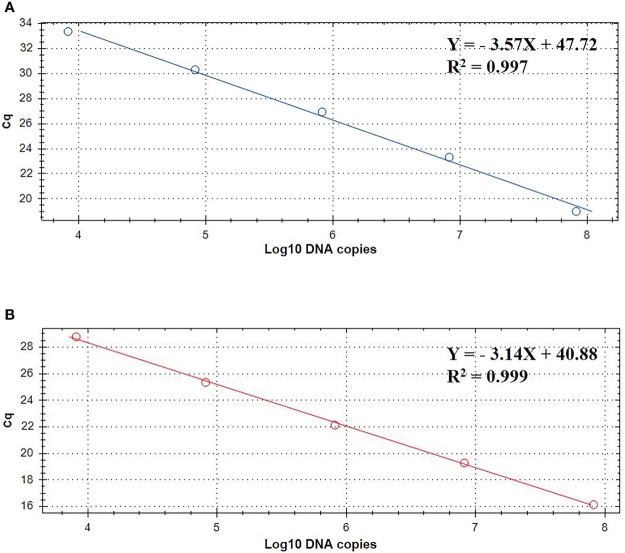
TaqMan standard curve of 10- fold dilutions of linearized plasmids pMD-gdh **(A)** and pMD-apxIVA **(B)** ranging from 10^3^ to 10^7^ DNA copies.

The standard curve of the TaqMan real-time PCR assay were determined using the recombinant plasmid standards pMD-gdh and pMD-apxIVA. The copy numbers of recombinant plasmid standards pMD-gdh and pMD-apxIVA were 9.343×10^10^ copies/μL and 8.644×10^10^ copies/μL, respectively. The standard curve established with the recombinant plasmids is depicted in **Figure 2**. In the gradient concentration range of 10^3^ copies/μL to 10^7^ copies/μL, the copy number of *S. suis* recombinant plasmid has a good linear relationship with the Cq value. The linear equation is Y=-3.57X+47.72, and the amplification efficiency is 95.9% with R^2^ value 0.997. The copy number of *A. pleuropneumoniae* recombinant plasmid also had a good linear relationship with the Cq value. The linear equation is Y=-3.14X+40.88, the amplification efficiency is 104.4%, and the R^2^ value is 0.999.

Since standard curves are a vital part of real-time PCR analysis, their accuracy and precision must be confirmed. In this study, the standard curve slopes are -3.57, -3.14 for *S. suis* and *A. pleuropneumoniae*, respectively. Moreover, the efficiencies are between 90% to 120%, suggesting the standard curve of this real-time PCR is considered reliable.

### Sensitivity and reproducibility

**Figure 3:**
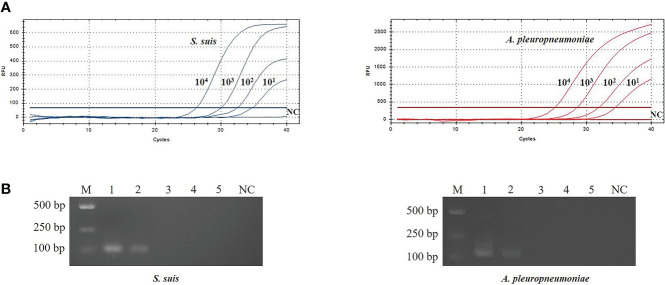
Evaluation of sensitivity for TaqMan real-time PCR **(A)** and conventional PCR **(B)**.The plasmid template dilution concentrations of *S. suis* and *A. pleuropneumoniae* were 7.534×10^4^~7.534×10^0^ copies/μL and 6.555×10^4^~6.555×10^0^ copies/μL, respectively. M: DL2000 Marker; B1-5: 10^4^ copies/μL-10^0^ copies/μL plasmid standards; NC, Negative control.

The sensitivity of TaqMan real-time PCR was analyzed using 10-fold serial dilutions of recombinant plasmids (pMD-gdh and pMD- apxIVA) DNA (10^0^ copies/μL~10^4^ copies/μL). The limit of detection using TaqMan real-time PCR was 10 copies/μL (**Figure 3**), while the limit of detection using conventional PCR is 10^3^ copies/μL (**Figure 3**). Traditional bacterial isolation and identification tests were used to detect the same samples, and the results were consistent with the results of conventional PCR, and all showed true positive. This result indicates that the sensitivity of TaqMan real-time PCR is 100 times that of conventional PCR.

In order to evaluate the reproducibility of TaqMan PCR, the serially diluted recombinant plasmids (pMD-gdh and pMD-apxIVA) were repeatedly tested every other week. As shown in **Table 2**, the coefficient of variation (CV) of pMD-gdh and pMD-apxIVA in the inter-assay and intra-assay were less than 3%, respectively. The results showed that the TaqMan real-time PCR method established in this study had good reproducibility.

**Table 2:** Evaluation of reproducibility of TaqMan real-time PCR.

Plasmid standard	Concentration (copies/μL)	Inter-assay (Cq)		Intra-assay (Cq)	
		Mean ± *SD*	CV (%)	Mean ± *SD*	CV (%)
pMD-gdh	7.534×10^6^	16.45 ± 0.13	0.82	16.56 ± 0.19	1.02
	7.534×10^5^	19.67 ± 0.11	0.56	19.56 ± 0.40	1.67
	7.534×10^4^	22.13 ± 0.13	0.52	22.36 ± 0.46	1.99
pMD-apxIVA	6.555×10^6^	19.79 ± 0.29	1.09	18.66 ± 0.56	2.01
	6.555×10^5^	21.44 ± 0.15	0.52	21.88 ± 0.22	1.09
	6.555×10^4^	24.67 ± 0.17	0.56	24.57 ± 0.29	1.23

### Calibration curves

**Figure 4:**
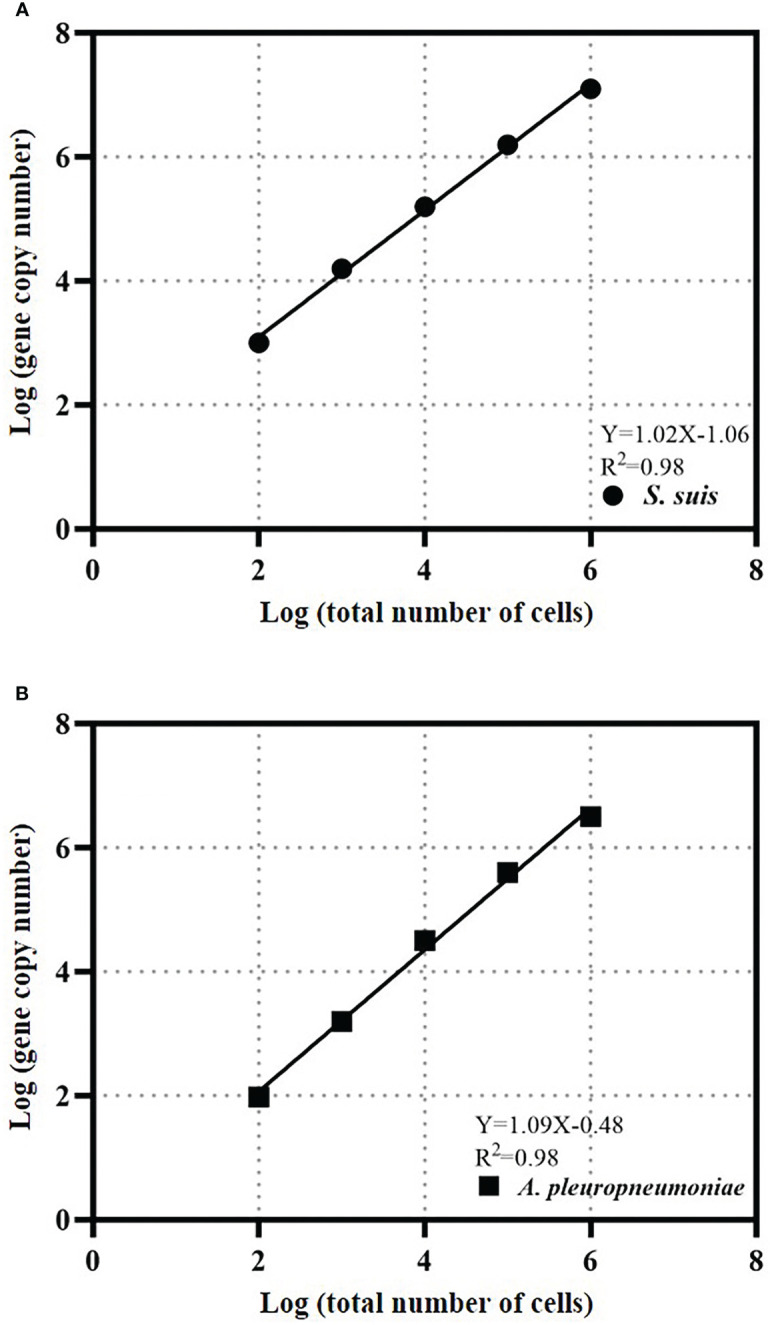
TaqMan real-time PCR estimates gene copy abundance and bacterial abundance of *S. suis* **(A)** or *A. pleuropneumoniae* **(B)**. The data is expressed as an average with 95% confidence interval band (n = 3).

Calibration curves for bacterial quantity and gene copy number were created for absolute quantification of cells. The calibration curve for *S. suis* bacteria was Y=1.02X-1.06 and the R^2^ value was 0.98 (**Figure 4**). The *A. pleuropneumoniae* concentration calibration curve was Y=1.09X-0.48 and the R^2^ value was 0.98 (**Figure 4**).

The cell calibration curve can avoid deviations that may be caused by the gene copy number or DNA extraction efficiency of each bacterium. The slopes of the cell calibration curves of *S. suis* and *A. pleuropneumoniae* are close to 1, which indicates that the gene copy number increases proportionally to the bacterial concentration in the test range, and the gene copy number of each bacterium does not change. The R^2^ values that the *gdh* gene of *S. suis* and copy number of *apx*IVA gene of *A. pleuropneumoniae* can be used to quantify bacterial quantity.

### Quantification of *S. suis* and *A. pleuropneumoniae* in clinical samples

A total of 45 clinical samples were used for evaluating the real-time PCR assay developed in this study. The suspected samples were analyzed by both TaqMan real-time PCR and conventional PCR. The results are presented as the percentage of positive samples. As reported in **Table 3**, the positive rate of TaqMan real-time PCR was 46.6% (21/45), including 13.3% (6/45) of *S. suis* infection, 26.7% (12/45) of *A. pleuropneumoniae* infection and 6.7% (3/45) of *S. suis* and *A. pleuropneumoniae* mixed infection. However, the positive rate of conventional PCR was 40% (18/45), including 13.3% (6/45) of *S. suis* infection, 20% (9/45) of *A. pleuropneumoniae* infection and 6.7% (3/45) of *S. suis* and *A. pleuropneumoniae* mixed infection. Traditional bacterial isolation and PCR identification tests were used to detect the same samples, and the results were consistent with TaqMan real-time PCR. The sensitivity of TaqMan real-time PCR was higher than that of conventional PCR.

**Table 3:** TaqMan real-time PCR and conventional PCR test results.

Detection method	Total number of samples	Positive samples				Positive rate (%)
		Positive rate of *S. suis* (%)	Positive rate of *A. pleuropneumoniae* (%)	Positive rate of Mixed (%)	Total	
TaqMan real-time PCR	45	13.3% (6)	26.7% (12)	6.7% (3)	21	46.6%
conventional PCR	45	13.3% (6)	20% (9)	6.7% (3)	18	40%

### Quantification of *S. suis* and *A. pleuropneumoniae* in biofilm samples

**Figure 5:**
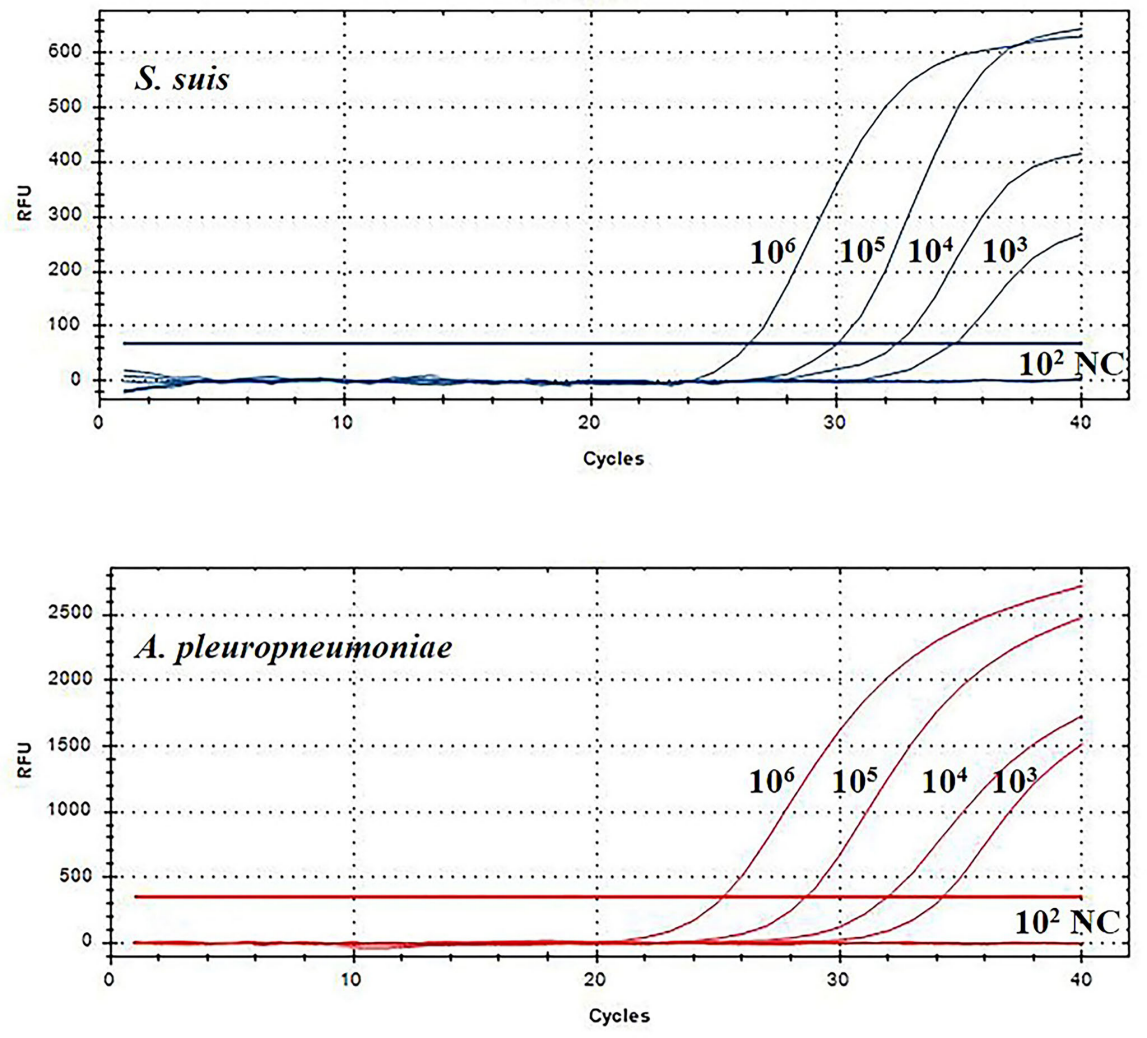
TaqMan real-time PCR analysis of biofilm sensitivity. The biofilm dilution multiple of *S. suis* and *A. pleuropneumoniae* was 10^2^-10^6^ CFUs/mL; NC, Negative control.

**Figure 6:**
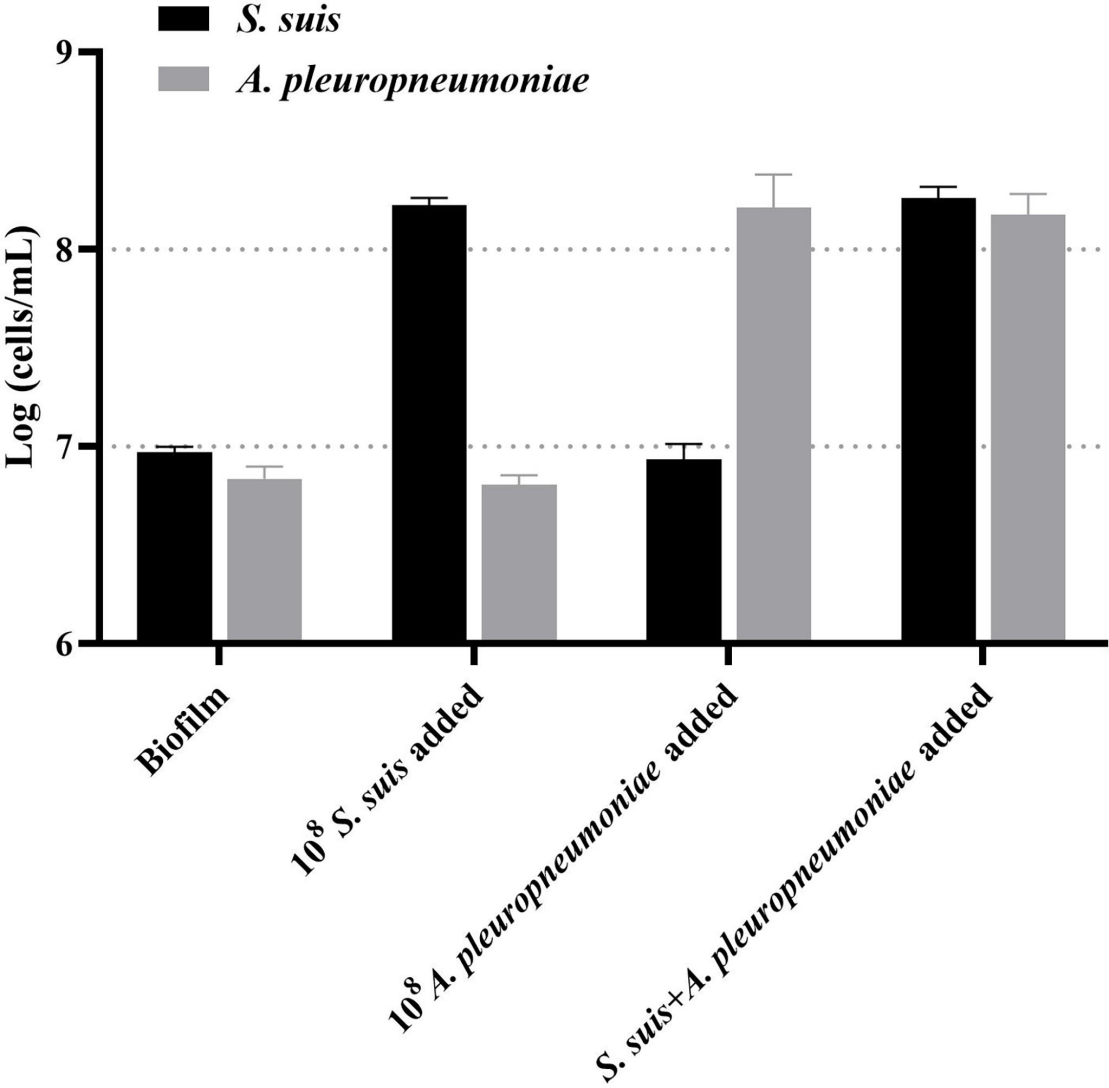
TaqMan real-time PCR analysis of biofilm samples, including: no added planktonic bacteria, 10^8^ CFUs/mL *S. suis*, 10^8^ CFUs/mL *A. pleuropneumoniae*, or the two bacterial species; data are expressed as mean ± standard deviation (n = 3).

Then, this TaqMan real-time PCR assay were also used for quantification of *S. suis* and *A. pleuropneumoniae* in biofilm system. For this purpose, a known amount of *S. suis* and *A. pleuropneumoniae* were added to a biofilm sample, and then amplified to assess the performance of the designed TaqMan real-time PCR assays in a biofilm system. The minimum detection limit of *S. suis* and *A. pleuropneumoniae* biofilm was 10^3^ CFUs/mL (**Figure 5**). The TaqMan real-time PCR assays revealed the initial abundance of *S. suis* and *A. pleuropneumoniae* in the biofilm to be approximately 9.56×10^6^ copies/mL and 7.89×10^6^ copies/mL, respectively. In the sample with 10^8^ CFUs/mL of *S. suis* added to the biofilm, 3.37×10^8^ copies/mL *S. suis* was detected with no detection of any additional *A. pleuropneumoniae* compared to the initial abundance in the biofilm (*P > 0.05*) (**Figure 6**). 10^8^ CFUs bacterial of *A. pleuropneumoniae* were added to the biofilm sample, and 2.89×10^8^ copies/mL *A. pleuropneumoniae* was detected. The addition of 10^8^ CFUs/mL *S. suis* and 10^8^ CFUs/mL *A. pleuropneumoniae* in biofilm samples gave the very similar results as when added alone (*P> 0.05*), which were 4.88×10^8^ copies/mL and 1.56×10^8^ copies/mL, respectively. It was thus concluded that each bacterium could be accurately quantitative in a biofilm sample.

## Discussion

In recent years, respiratory diseases constitute one of the most important health issues affecting the swine industry worldwide. Polymicrobial respiratory diseases have also been threatening the swine industry worldwide. *S. suis* and *A. pleuropneumoniae* are important pathogens associated with PRDC. Their co-occurrence in the same site of infection has been frequently reported (Opriessnig et al., 2011), resulting in serious mixed infection of pigs, increasing the lethality, which has seriously affected the development of swine industry. Although the traditional detection methods such as PCR and ELISA provide a reliable method to distinguish *S. suis* and *A. pleuropneumoniae* respectively, it is labor intensive, complex and time consuming (Srinivasan et al., 2016; Teshima et al., 2017; Xia et al., 2017; Srijuntongsiri et al., 2022). Nowadays, reducing cost and time of experiment is critical for pathogen detection. Therefore, the rapid and accurate detection method has the potential to be of great significance for the diagnosis and preventing the co-infection of *S. suis* and *A. pleuropneumoniae*. Through literature review and practice, we found that real-time PCR is a powerful and easy to use technology (Kralik and Ricchi, 2017). Thus, this study developed a TaqMan real-time PCR assay that simultaneously detected and differentiated *S. suis* and *A. pleuropneumoniaeare*, which exhibited efficiently identification in clinical samples and mixed biofilms.

In this study, a TaqMan real-time PCR assay targeting the *gdh* gene of *S. suis* and the *apx*IVA gene of *A. pleuropneumoniae* was designed and validated. First, the *gdh* gene of *S. suis* and the *apx*IVA gene of *A. pleuropneumoniae* were sequenced to confirm the identity of the two bacterial species used for this study. Following this step, the confirmed gene sequences were used to design bacterial species-specific real-time PCR primers and probes. Then, the real-time PCR reaction system was established and optimized, and standard curves for the real-time PCR assays were developed. Finally, the specificity, sensitivity and reproducibility of the assay were evaluated, and the assay was validated by cross-reactivity tests and measuring known concentrations of each bacterium in biofilm samples. The results showed that the method has high sensitivity, high-throughput and strong specificity. This method can accurately quantify not only *S. suis* and *A. pleuropneumoniae* in mixed infection, but also *S. suis* and *A. pleuropneumoniae* in mixed biofilm samples. In order to prevent the long-term coexistence of *S. suis* and *A. pleuropneumoniae* resulting in the formation of biofilms, it provides a new technical means for the rapid detection of the co-infection of *S. suis* and *A. pleuropneumoniae* (Wang et al., 2020). The results of this study serve as a proof of concept and exemplifies successful application of TaqMan real-time PCR for the specific quantification of *S. suis* and *A. pleuropneumoniae* in mixed-community biofilms *in vitro* for the first time. At the same time, by quantifying and comparing the biomass of each species in a mixed biofilm, we can better understand the interaction between the species in the mixed biofilm. With the demand for faster and more accurate detection of clinical materials, this TaqMan real-time PCR detection method may become an invaluable tool.

The methods commonly used to study mixed biofilms have several limitations. For example, crystal violet staining and viable cell count have poor reproducibility when analyzing multi-species biofilms due to bacterial coaggregation (Stepanovic et al., 2000; Martinez and Casadevall, 2007). The method combining laser confocal scanning microscopy (CLSM) and fluorescence *in situ* hybridization (FISH) is only suitable for qualitative studies of biofilm, but is not suitable for high-throughput comparative screening studies (Bridier et al., 2014; Reichhardt and Parsek, 2019). Accurate detection of *S. suis* and *A. pleuropneumoniae* in biofilm samples showed that the designed TaqMan real-time PCR assay is an effective method for quantifying biofilms. On the contrary of the crystal violet staining method, this procedure can quantify each species in a mixed biofilm. Live cell counting can also quantify individual species in a mixed biofilm, but it has many influencing factors. For example, live bacteria are affected by sample processing, and consequently there is an error in the counting process. The method established in this study also has some limitations. One is that it cannot distinguish between live and dead bacteria. The second is that the method established in this study may not be applicable to all strains. Consequently, it is very important that various methods can verify and complement each other when studying mixed biofilms. We can use these three methods of crystal violet staining, living cell counting and TaqMan real-time PCR together to complement each other.

Further studies are necessary in order to fully highlight the impact of mixed bacterial infections detection in disease prevention. In the future, our research group will focus on increase the number of primers, optimize the reaction conditions, improve the amplification efficiency and reduce the detection limit. TaqMan real-time PCR is combined with other molecular biology techniques to further improve the detection sensitivity, detection range and detection specificity. This qPCR technique can be widely used for the detection of bacterial mixed infection, it may represent a reference for the rapid detection of bacterial pathogens in the breeding industry, and finally it lays a foundation for the prevention and control of porcine respiratory diseases.

## Data availability statement

The original contributions presented in the study are included in the article/supplementary files. Further inquiries can be directed to the corresponding authors.

## Author contributions

YW and SW designed the study and approved the manuscript. LY, LS, DG, MG and MJ developed the multiplex PCR method, analyzed data and drafted the manuscript. MJ, HW and QF contribute to reagents/materials/analysis tools. All authors read and approved the final manuscript.

## Funding

This work was supported by the National Natural Science Foundation of China (32172852, 31902309 and 31972654), the National Key Research and Development Program of China (2018YFD0500104), and Excellent Youth Foundation of He’nan Scientific Committee (222300420005), the Scientific and Technical Innovation Project of the Chinese Academy of Agricultural Sciences (SHVRI-ASTIP-2014-8).

## Conflict of interest

The authors declare that the research was conducted in the absence of any commercial or financial relationships that could be construed as a potential conflict of interest.

## Publisher’s note

All claims expressed in this article are solely those of the authors and do not necessarily represent those of their affiliated organizations, or those of the publisher, the editors and the reviewers. Any product that may be evaluated in this article, or claim that may be made by its manufacturer, is not guaranteed or endorsed by the publisher.

## References

[B1] Bridier A. Briandet R. Bouchez T. Jabot F. (2014). A model-based approach to detect interspecific interactions during biofilm development. Biofouling 30 (7), 761–771. doi: 10.1080/08927014.2014.923409 24963685

[B2] Burmolle M. Ren D. Bjarnsholt T. Sorensen S. J. (2014). Interactions in multispecies biofilms: Do they actually matter? Trends Microbiol. 22 (2), 84–91. doi: 10.1016/j.tim.2013.12.004 24440178

[B3] Cao H. Shockey J. M. (2012). Comparison of TaqMan and SYBR green qPCR methods for quantitative gene expression in tung tree tissues. J. Agric. Food Chem. 60 (50), 12296–12303. doi: 10.1021/jf304690e 23176309

[B4] Costerton J. W. Stewart P. S. Greenberg E. P. (1999). Bacterial biofilms: a common cause of persistent infections. Science 284 (5418), 1318–1322. doi: 10.1126/science.284.5418.1318 10334980

[B5] Elias S. Banin E. (2012). Multi-species biofilms: living with friendly neighbors. FEMS Microbiol. Rev. 36 (5), 990–1004. doi: 10.1111/j.1574-6976.2012.00325.x 22229800

[B6] Figueiredo A. M. S. Ferreira F. A. Beltrame C. O. Cortes M. F. (2017). The role of biofilms in persistent infections and factors involved in ica-independent biofilm development and gene regulation in *Staphylococcus aureus* . Crit. Rev. Microbiol. 43 (5), 602–620. doi: 10.1080/1040841X.2017.1282941 28581360

[B7] Harriott M. M. Noverr M. C. (2009). *Candida albicans* and *Staphylococcus aureus* form polymicrobial biofilms: Effects on antimicrobial resistance. Antimicrob. Agents Chemother. 53 (9), 3914–3922. doi: 10.1128/AAC.00657-09 19564370 PMC2737866

[B8] Horter D. Chang C. C. Pogranichnyy R. Zimmerman J. Yoon K. J. (2001). Persistence of porcine reproductive and respiratory syndrome in pigs. Adv. Exp. Med. Biol. 494, 91–94. doi: 10.1007/978-1-4615-1325-4_14 11774551

[B9] Kirakodu S. S. Govindaswami M. Novak M. J. Ebersole J. L. Novak K. F. (2008). Optimizing qPCR for the quantification of periodontal pathogens in a complex plaque biofilm. Open Dent. J. 2, 49–55. doi: 10.2174/1874210600802010049 19088882 PMC2581537

[B10] Kolter R. Greenberg E. P. (2006). Microbial sciences: the superficial life of microbes. Nature 441 (7091), 300–302. doi: 10.1038/441300a 16710410

[B11] Kralik P. Ricchi M. (2017). A basic guide to real time PCR in microbial diagnostics: definitions, parameters, and everything. Front. Microbiol. 8. doi: 10.3389/fmicb.2017.00108 PMC5288344 28210243

[B12] Martinez L. R. Casadevall A. (2007). *Cryptococcus neoformans* biofilm formation depends on surface support and carbon source and reduces fungal cell susceptibility to heat, cold, and UV light. Appl. Environ. Microbiol. 73 (14), 4592–4601. doi: 10.1128/AEM.02506-06 17513597 PMC1932807

[B13] Mullebner A. Sassu E. L. Ladinig A. Frombling J. Miller I. Ehling-Schulz M.. (2018). *Actinobacillus pleuropneumoniae* triggers IL-10 expression in tonsils to mediate colonisation and persistence of infection in pigs. Vet. Immunol. Immunopathol. 205, 17–23. doi: 10.1016/j.vetimm.2018.10.008 30458998

[B14] Opriessnig T. Gimenez-Lirola L. G. Halbur P. G. (2011). Polymicrobial respiratory disease in pigs. Anim. Health Res. Rev. 12 (2), 133–148. doi: 10.1017/S1466252311000120 22152290

[B15] Reichhardt C. Parsek M. R. (2019). Confocal laser scanning microscopy for analysis of *Pseudomonas aeruginosa* biofilm architecture and matrix localization. Front. Microbiol. 10. doi: 10.3389/fmicb.2019.00677 PMC6454187 31001240

[B16] Rendueles O. Ghigo J. M. (2012). Multi-species biofilms: how to avoid unfriendly neighbors. FEMS Microbiol. Rev. 36 (5), 972–989. doi: 10.1111/j.1574-6976.2012.00328.x 22273363

[B17] Segura M. Fittipaldi N. Calzas C. Gottschalk M. (2017). Critical *Streptococcus suis* virulence factors: Are they all really critical? Trends Microbiol. 25 (7), 585–599. doi: 10.1016/j.tim.2017.02.005 28274524

[B18] Srijuntongsiri G. Mhoowai A. Samngamnim S. Assavacheep P. Bosse J. T. Langford P. R.. (2022). Novel DNA markers for identification of *actinobacillus pleuropneumoniae* . Microbiol. Spectr. 10 (1), e0131121. doi: 10.1128/spectrum.01311-21 34985298 PMC8729771

[B19] Srinivasan V. McGee L. Njanpop-Lafourcade B. M. Moisi J. Beall B. (2016). Species-specific real-time PCR assay for the detection of *Streptococcus suis* from clinical specimens. Diagn. Microbiol. Infect. Dis. 85 (2), 131–132. doi: 10.1016/j.diagmicrobio.2016.02.013 27041105

[B20] Stepanovic S. Vukovic D. Dakic I. Savic B. Svabic-Vlahovic M. (2000). A modified microtiter-plate test for quantification of staphylococcal biofilm formation. J. Microbiol. Methods 40 (2), 175–179. doi: 10.1016/s0167-7012(00)00122-6 10699673

[B21] Suzuki N. Yoshida A. Nakano Y. (2005). Quantitative analysis of multi-species oral biofilms by TaqMan real-time PCR. Clin. Med. Res. 3 (3), 176–185. doi: 10.3121/cmr.3.3.176 16160072 PMC1237159

[B22] Teshima K. Lee J. To H. Kamada T. Tazumi A. Hirano H.. (2017). Application of an enzyme-linked immunosorbent assay for detection of antibodies to *Actinobacillus pleuropneumoniae* serovar 15 in pig sera. J. Vet. Med. Sci. 79 (12), 1968–1972. doi: 10.1292/jvms.17-0374 29070770 PMC5745173

[B23] Wang Y. Gong S. Dong X. Li J. Grenier D. Yi L. (2020). *In vitro* mixed biofilm of *Streptococcus suis* and *Actinobacillus pleuropneumoniae* impacts antibiotic susceptibility and modulates virulence factor gene expression. Front. Microbiol. 11. doi: 10.3389/fmicb.2020.00507 PMC7179662 32373078

[B24] Wang L. Li Y. Wang L. Zhang H. Zhu M. Zhang P.. (2018). Extracellular polymeric substances affect the responses of multi-species biofilms in the presence of sulfamethizole. Environ. Pollut. 235, 283–292. doi: 10.1016/j.envpol.2017.12.060 29291528

[B25] Weinert L. A. Chaudhuri R. R. Wang J. Peters S. E. Corander J. Jombart T.. (2015). Genomic signatures of human and animal disease in the zoonotic pathogen *Streptococcus suis* . Nat. Commun. 6, 6740. doi: 10.1038/ncomms7740 25824154 PMC4389249

[B26] Whelan J. A. Russell N. B. Whelan M. A. (2003). A method for the absolute quantification of cDNA using real-time PCR. J. Immunol. Methods 278 (1-2), 261–269. doi: 10.1016/S0022-1759(03)00223-0 12957413

[B27] Xia X. J. Wang L. Shen Z. Q. Qin W. Hu J. Jiang S. J.. (2017). Development of an indirect dot-PPA-ELISA using glutamate dehydrogenase as a diagnostic antigen for the rapid and specific detection of *Streptococcus suis* and its application to clinical specimens. Antonie. Van. Leeuwenhoek. 110 (4), 585–592. doi: 10.1007/s10482-016-0825-z 28058577

[B28] Yao H. Chen G. Lu C. (1999). Identification of isolates of swine streptococcus in jiangsu province during 1998. J. Nanjing. Agric. Univ. 22 (2), 67–70. doi: CNKI:SUN:NJNY.0.1999-02-015

